# The association between dietary total antioxidant capacity and hearing loss: results from the Tehran employees Cohort Study

**DOI:** 10.1186/s12889-024-18108-6

**Published:** 2024-03-15

**Authors:** Batoul Ghosn, Leila Azadbakht, Mohammad Reza Monazzam Esmaeilpour, Ahmad Esmaillzadeh

**Affiliations:** 1 Department of Community Nutrition, School of Nutritional Sciences and Dietetics, Tehran University of Medical Sciences, P.O. Box 14155-6117, Tehran, Iran; 2 Department of Community Nutrition, School of Nutrition and Food Science, Isfahan University of Medical Sciences, Isfahan, Iran; 3 Obesity and Eating Habits Research Center, Endocrinology and Metabolism Molecular- Cellular Sciences Institute, Tehran University of Medical Sciences, Tehran, Iran; 4 Department of Occupational Hygiene, School of Public Health, Center for Air Pollution Research (CAPR), Institute for Environmental Research (IER), Tehran University of Medical Sciences, Tehran, Iran

**Keywords:** Dietary total antioxidant capacity, Hearing loss, Tehran employees Cohort Study, Young men, Antioxidant intake

## Abstract

**Background:**

Despite numerous studies that have explored the association between individual antioxidants or specific combinations and the risk of hearing loss, there is lack of information regarding the relationship between dietary total antioxidant capacity (dTAC) and hearing loss. The conflicting results on this association further highlight the need for more research in this area. This study aims to investigate the association between overall dietary antioxidant intake and the risk of hearing loss among Iranian adults.

**Methods:**

This cross-sectional study recruited 3443 adult participants aged between 19 and 67 years (with an average age of 41.4 years ± 8.8) who were employed at Tehran University of Medical Sciences in Iran. Participants underwent dietary assessment using a validated Food Frequency Questionnaire (FFQ). The hearing status of each participant was evaluated by a licensed audiologist in a soundproof room, using diagnostic audiometry that adhered to American National Standards specifications and followed standard audiometric clinical procedures. The dietary total antioxidant capacity (dTAC) was calculated using the Ferric Reducing-Antioxidant Power (FRAP) values.

**Results:**

43.6% of male participants had hearing loss, while 26.8% among female participants. After accounting for various confounding factors, no significant association was observed between higher levels of dTAC and reduced odds of hearing loss in the overall population. However, among men under the age of 40, higher levels of dTAC were associated with decreased odds of hearing loss, even after adjusting for several covariates (OR: 0.56, 95% CI: 0.31–1.02, Ptrend = 0.02). This relationship was not evident in men over 40 years of age or among women.

**Conclusion:**

The study found an inverse relationship between higher antioxidant intake in the diet and lower odds of hearing loss, specifically among men who were 40 years old or younger. However, this relationship was not observed in the overall population or among women. Additional prospective studies are necessary to validate these results.

## Introduction

Onset of hearing impairment in adults is considered as the fifth leading cause of years lived with disability [1]. According to the World Health Organization (WHO), there are 360 million people who suffer from disabling hearing impairment worldwide [2]. Data on the prevalence of hearing impairment in Iran is scarce. In 2008, the WHO estimated the prevalence of mild hearing impairment in the Middle East region at 16.4% for males and 12.8% for females aged more than 15 years old [3]. The prevalence of hearing impairment is greatest in low and middle-income regions [3] rising the need for more investigation in this field in these regions and particularly in Iran, to assess its causes and study possible interventions.

A healthy diet is an important modifiable risk factor for hearing loss. Moderate consumption of fish, whole grains and moderate intake of alcohol [4–7] as well as vegetables and nuts [8] has been associated with lower hearing thresholds. In addition, some studies have detected the interaction between different food groups such as fish and carbohydrate consumption with hearing thresholds [4, 9, 10], highlighting the importance to study the effects of dietary patterns as well.

Previous studies have shown that antioxidant supplementation might help improving hearing loss [11–19]. Previous cross-sectional studies [20–23], clinical trials [15, 19] and animal studies that have examined the role of antioxidants in hearing loss had demonstrated a slowing down effect on the progression of hearing impairment [24]; however, some prospective studies failed to reach such a conclusion [25]. It seems that antioxidants might prevent lipid peroxidation in the cell by scavenging the single oxygen [26] or by reducing and reacting with peroxyl radicals [27], through which they prevent hearing impairment.

One of the main causes of hearing loss is oxidative stress, which occurs when the production of reactive oxygen species (ROS) exceeds the capacity of the antioxidant defense system [28]. Free radicals are damaging molecules known to cause cell death, and they can be particularly harmful to the sensory cells in the ears [29]. Antioxidants can prevent free radical damage by binding to these molecules and rendering them harmless [30]. This protective effect of antioxidants may help to prevent or slow the progression of hearing loss [24].

Antioxidants are substances that can scavenge ROS and protect the inner ear from oxidative damage [31]. Several studies have shown that antioxidants can prevent or delay the onset of age-related hearing loss by enhancing the endogenous antioxidant enzymes [32], reducing the inflammation and apoptosis [24], and improving the mitochondrial function and blood flow in the cochlea [33].

Given the high prevalence of hearing loss worldwide and specifically in developing countries, and the limited evidence in existing literature linking diet and psychological factors to hearing loss, this study investigated the association between dietary total antioxidant capacity and psychological factors and hearing loss, with the main objective of assessing the interaction between these two independent factors.

## Materials and methods

### Participants

This study used data from a sample of employees at Tehran University of Medical Sciences. The cohort began in 2018 and is still ongoing, collecting information from over 4,000 employees. Participants with specific dietary restrictions or non-feasible energy intake, a history of head injury, major ear operations, asymmetrical or conductive hearing loss, significant recreational noise exposure, uncontrolled systemic illnesses, chronic middle ear pathology, current ear problems, or the use of a hearing aids or cochlear implants were excluded from the analysis. Participants were included if they have a clinical diagnosis of conductive, sensorineural, or mixed hearing loss of varying severity. Every individual involved in this study gave their consent in writing. The Medical Ethics Committee of Tehran University of Medical Sciences, Tehran, Iran, granted approval for the entire study. Additionally, the Ethics committee separately approved this specific project under the ethical code IR.TUMS.MEDICINE.REC.1401.480. The questionnaire thoroughly explained the aim of the study, and participation in answering the questions was entirely voluntary. We guaranteed all participants that their information would remain anonymous and confidential.

### Assessment of dietary intakes

The study assessed dietary intake using a validated, self-administered 144-item food frequency questionnaire (FFQ) that covers a variety of foods such as fruits, vegetables, grains, mixed dishes, dairy products, and other foods and beverages. Participants indicated how often they consume these foods using nine multiple-choice frequency response categories. Nutrient intake was calculated by using the US Department of Agriculture’s national nutrient databank.

In our study, we employed a thorough approach to measure the Dietary Total Antioxidant Capacity (dTAC). We began by examining all food items reported in the Food Frequency Questionnaire (FFQ) that have established Ferric Reducing-Antioxidant Power (FRAP) values. These food items spanned a broad spectrum of categories, including fruits, vegetables, grains, meats, dairy products, and beverages. The FRAP value, which quantifies the capacity of dietary antioxidants in the food to reduce ferric ions to ferrous ions, was used to calculate the contribution of each food item to the dTAC. This value is expressed as millimoles per 100 g of food (mmol/100 g) [34, 35]. We retrieved the dTAC based on the FRAP values from published databases [34]. For food items with similar properties, such as various types of bread, we computed an overall mean dTAC value. In cases where a food item lacked TAC data, we assigned the value of the most similar food item. Finally, we calculated the dTAC for all participants by multiplying the corresponding FRAP values by the consumption frequency of each food item and then summing these values. This method enabled us to estimate the dietary total antioxidant capacity of each participant’s diet in our study.

The general formula for calculating dTAC is:

$$ \text{dTAC}=\sum _{i=1}^{n}({\text{FRAP}}_{i}\times {\text{Consumption}}_{i})$$

Where:

- dTAC is the Dietary Total Antioxidant Capacity.
- FRAPi is the Ferric Reducing-Antioxidant Power value of the i-th food item.
- Consumptioni is the frequency of consumption of the i-th food item.
- The sum is over all n food items consumed.

The validity and reliability of a dish-based semi-quantitative food frequency questionnaire (FFQ) for assessing dietary intake was previously established [36]. The study examined the validity of the FFQ using a sample of 282 participants and using six 24-hour dietary recalls and biomarkers (blood and urine samples) as the gold standard. All participants completed two separate FFQs with a 6-month interval in summer and winter seasons. To assess the validity, the study used energy-adjusted de-attenuated correlation coefficients (CC) and cross-classification analyses, and for reliability, intra-class correlation coefficients (ICC) were used. The results showed that the de-attenuated correlation coefficients of the FFQ and the 24-hour recalls varied between 0.32 and 0.88 (Mean = 0.59). The de-attenuated correlation coefficients between the FFQ and the plasma levels of retinol was 0.58 and between the FFQ and beta-carotene was 0.40 (*P* < 0.05). Only 5% of the participants were classified in opposite quartiles while cross-classification into same or adjacent quartiles varied between 67.7% (for dietary fiber) and 82.6% (for Thiamin). Nutrients correlation coefficients ranged between the two FFQs from 0.4 to 0.85. All these findings demonstrate that the questionnaire is valid and reliable in measuring long-term dietary intake.

### Assessment of hearing loss

A licensed audiologist assessed the hearing status of participants in a soundproof room with diagnostic audiometer meeting the American National Standards specifications and by obeying the standard audiometric clinical procedures [37]. First, hearing thresholds were acquired in frequencies of 0.25, 0.5, 1, 2, and 4 kHz for both ears through air conduction and bone conduction. Air conduction thresholds were given at eight intervals ranging from 0.25 to 8 kHz for each ear individually. Hearing function was measured as an improved Pure Tone Average through 1, 2 and 4 kHz. To prevent false measurement, covering noise was applied to dominate the non-test ear as the other ear was examined to prevent cross-hearing phenomenon at minimum interaural attenuation level of 40 dB. In the measurement of bone conduction thresholds, a masking procedure was used using ABC methods [38]. Using the initial audiogram data, the grade of hearing, loss was then determined as defined by the Japanese Ministry of Health and Welfare guidelines [39]. The severity of hearing loss was established by a mean hearing loss in frequencies of 250 to 4000 Hz. Up to 40 dB hearing loss was classified as mild, 41 to 70 dB hearing loss was classified as moderate, and 71 dB hearing loss and more was classified as severe. Middle ear health status was assessed by tympanometry. Participants who did not respond in their audiograms to at least one frequency were classified as nonresponses. Reliability of participants response was done by testing the 1 kHz frequency twice in each ear and unreliable response was defined by audiograms having a difference of 10-dB or more between the 2 tests.

### Assessment of biochemical indicators

To assess biochemical indicators, blood samples were taken from all participants after they had fasted overnight for 12–14 h. The samples were collected between 8:00 and 9:30 am. The blood was then centrifuged for 10 min at 3000 rpm to separate the plasma, which was then frozen for future analysis. Enzymatic colorimetric method was used to measure fasting plasma glucose and serum triglyceride. Phosphor tungstic acid was used to determine the concentrations of serum high-density lipoprotein cholesterol (HDL-C) after removing apolipoprotein B-containing lipoproteins. The analyses were conducted using commercial kits from Pars Azmoon Inc. based in Tehran, Iran.

### Assessment of blood pressure

The blood pressure of participants was measured three times using a standardized mercury sphygmomanometer after they had rested for 20 min and were in the sitting position. The measurements were taken from the right arm. Participants were given a rest period of 2–4 h between each measurement. The average of the three measurements was considered as the participant’s blood pressure [40].

### Assessment of ototoxic medications

Ototoxic medication use was defined as self-reported use of aminoglycoside, nonsteroidal anti-inflammatory drugs. loop diuretics and antineoplastic drugs.

### Assessment of anthropometric measures

The weight of the participants was measured to the nearest 0.1 kg using a digital scale, and the height and waist circumference were measured to the nearest 0.1 cm. Participants were weighed while wearing light clothing and no shoes. Height was measured using a tape measure with participants standing without shoes. The waist circumference was measured using a soft tape meter without applying pressure, at the midpoint between the lower rib margin and iliac crest. Body mass index (BMI) was calculated by dividing the weight in kilograms by the square of the height in meters.

### Assessment of other variables

The assessment of physical activity was done by calculating the metabolic equivalent-minute per week (METS-min/week) and using the short form of International Physical Activity Questionnaire (IPAQ). The IPAQ has been previously validated in other studies [41]. METS scores for walking (at least 10 min), vigorous and moderate intensity activities were multiplied by the time spent on these activities by the participants and their frequency in the past week. The total MET-min/week was obtained by adding up the scores for different activities. In addition, a self-administered questionnaire was used to gather information on other covariates such as sex, age, smoking status, use of nutritional supplements, medications, and marital status.

### Statistical analysis

The purpose of the study was to examine the correlation between dietary total antioxidant capacity (dTAC) and the risk of hearing loss. The participants were separated into five groups based on their dTAC levels, and their attributes were compared using one-way analysis of variance (ANOVA) for continuous variables and chi-square test for categorical variables. The dietary intake of each participant was also compared across the quintiles using ANOVA. To assess the relationship between dTAC and the risk of hearing loss, the study utilized multivariable logistic regression for the entire population and another separate analysis for individuals under or over 40 years old and for each gender. At the age of 40, a person’s hearing abilities may begin to decline, which is why it is considered the age at which normal hearing may start to deteriorate [42]. Also, the likelihood of men experiencing hearing loss is two times greater than that of women [43] which is why we stratified by gender. The models were adjusted for various factors including smoking, physical activity, family history of hearing loss, history of ear related issues, hypertension and diabetes, and BMI. The study calculated the trend of odds ratios across the increasing quintiles of dTAC and treated the quintiles as ordinal variables in the analysis. Results with a *p*-value greater than 0.05 were considered statistically significant. All data was analysed using SPSS version 18.

## Results

Table 1 presents the general characteristics of the study participants, categorized into five quintiles based on their dietary total antioxidant capacity (dTAC) levels. Each quintile represents a range of dTAC levels, with Q1 having the lowest and Q5 the highest. The data, presented as mean ± standard deviation for continuous variables and count (percent) for categorical variables, show no significant differences across the quintiles for any of these factors. This suggests that the distribution of these characteristics is fairly uniform across different levels of dTAC intake. For instance, the average age of participants ranged from 41 to 42 years across all quintiles, with a *p*-value of 0.26 indicating no significant difference in age distribution among the groups. Similarly, the proportion of participants who were university graduates was around 71–73% across all quintiles, with a *p*-value of 0.79 suggesting no significant variation in education level based on dTAC intake. The same pattern was observed for other variables such as physical activity, BMI, and health conditions like hypertension and diabetes. This uniform distribution of characteristics across quintiles allows for a more focused examination of the potential effects of dTAC levels on the outcomes of interest in the study.

**Table 1:** General characteristics of study population across quintiles of total antioxidant capacity

Quintiles dietary total antioxidant capacity (mmol/d)						
Variables	Q1 (*n* = 686) (≤ 8.2)	Q2 (*n* = 688) (8.2–9.15)	Q3 (*n* = 687) (9.15–10.15)	Q4 (*n* = 689) (10.15–11.7)	Q5 (*n* = 688) (≥ 11.7)	*P*-value
Age (years)	40.71 ± 8.63	41.28 ± 8.86	41.63 ± 8.92	41.63 ± 8.92	41.54 ± 8.63	0.26
Physical Activity (MET-min/week)	547.02 ± 886.24	509.04 ± 840.63	438.26 ± 661.49	465.57 ± 652.21	509.08 ± 860.61	0.09
Married (%)	569 (82.8)	569 (82.7)	564 (82.0)	572 (83.1)	574 (83.7)	0.94
BMI (kg/m2)	26.68 ± 4.20	26.86 ± 5.59	26.75 ± 4.39	26.73 ± 5.13	26.57 ± 4.55	0.85
Educated (university graduated) (%)	497 (72.3)	505 (73.4)	487 (70.8)	488 (70.9)	497 (72.4)	0.79
Gender (women)	410 (59.7)	422 (61.3)	431 (62.6)	411 (59.7)	429 (62.4)	0.67
Social economic status (%)						0.94
Low	96 (14.0)	102 (14.8)	97 (14.1)	100 (14.5)	87 (12.7)	
Middle	552 (80.3)	555 (80.7)	555 (80.7)	556 (80.8)	562 (81.5)	
High	39 (5.7)	31 (4.5)	36 (5.2)	32 (4.7)	38 (5.5)	
Smoker (%)	56 (8.2)	72 (10.5)	54 (7.8)	62 (9.0)	59 (8.6)	0.46
Hypertension (%)	95 (13.9)	122 (18.2)	106 (15.6)	103 (15.1)	107 (15.6)	0.29
Diabetes (%)	60 (9.2)	62 (9.4)	44(7.9)	56 (8.3)	51 (7.2)	0.37
Family History HL (%)	59 (8.6)	55 (8.3)	72 (10.5)	69 (10.4)	54 (8.4)	0.31
Ototoxic Medication use (%)	56 (8.1)	58 (8.4)	59 (9.1)	43 (6.3)	54 (8.5)	0.5

Data are presented as Mean ± SD and count (percent)

Abbreviations: BMI Body Mass Index

*Obtained from the one-way analysis of variance (ANOVA) or Chi-square test, where appropriate

Table 2 provides a detailed breakdown of the dietary intakes of the study participants, categorized into five quintiles based on their dietary total antioxidant capacity (dTAC) levels. The table presents data on total energy intake, intake of various food groups (including protein, carbohydrates, and different types of fats), cholesterol, fiber, and several micronutrients. For each variable, the table shows the mean intake for participants in each quintile, with Q1 representing the lowest and Q5 the highest dTAC levels. The *p*-values indicate the statistical significance of the differences in intake across the quintiles. For instance, the total energy intake varied significantly across the quintiles, ranging from an average of 3402 kcal/d in Q1 to 3313 kcal/d in Q5, with a *p*-value of less than 0.001 indicating a statistically significant difference. Similarly, intake of protein, carbohydrates, and fats also varied significantly across the quintiles. For example, average protein intake ranged from 102 g/d in Q2 to 128 g/d in Q1 and Q5, with a *p*-value of less than 0.001. The table also presents data on the intake of various micronutrients, including magnesium, vitamins A, E, C, B12, D, folate, and beta-carotene. For all these nutrients, intake varied significantly across the quintiles. For instance, average vitamin C intake ranged from 103 mg/d in Q2 to 189 mg/d in Q5, with a *p*-value of less than 0.001. These findings suggest that dietary total antioxidant capacity is associated with significant variations in the intake of various nutrients among the study participants.

**Table 2:** Dietary intakes of study participants across quintiles of total dietary antioxidant capacity

Quintiles of dietary total antioxidant capacity (mmol/d)						
Variables	Q1 (*n* = 686) (≤ 8.2)	Q2 (*n* = 688) (8.2–9.15)	Q3 (*n* = 687) (9.15–10.15)	Q4 (*n* = 689) (10.15–11.7)	Q5 (*n* = 688) (≥ 11.7)	*P*-value
Total energy (kcal/d)	3402.35 ± 5154.31	2712.80 ± 863.56	2725.22 ± 937.08	2891.57 ± 1141.87	3312.71 ± 1494.54	< 0.001
**Food Groups (g)**						
Protein (g/d)	128.35 ± 167.21	102.66 ± 35.95	102.62 ± 36.2	109.50 ± 45.51	125.12 ± 59.26	< 0.001
Carbohydrate (g/d)	465.88 ± 510.15	385.57 ± 127.95	389.22 ± 140.59	410.85 ± 175.82	471.51 ± 219.12	< 0.001
Fat (g/d)	117.49 ± 284.25	87.52 ± 32.47	87.83 ± 35.99	94.54 ± 39.03	109.59 ± 58.94	< 0.001
SFA (g/d)	26.7 ± 45.85	20.96 ± 8.40	20.62 ± 8.47	22.62 ± 9.55	26.02 ± 14.13	< 0.001
MUFA (g/d)	26.72 ± 60.89	19.96 ± 7.81	19.94 ± 8.24	21.50 ± 8.21	25.14 ± 13.51	< 0.001
PUFA (g/d)	44.56 ± 140.51	32.21 ± 12.54	32.69 ± 14.88	34.65 ± 15.49	40.40 ± 22.89	0.001
Cholesterol (mg/d)	366.02 ± 487.23	278.89 ± 135.05	278.14 ± 143.03	291.45 ± 164.51	340.24 ± 270.43	< 0.001
Fiber (g/d)	19.25 ± 23.68	16.7 ± 5.24	18.42 ± 7.03	20.15 ± 8.43	26.05 ± 12.72	< 0.001
**Micronutrients**						
Magnesium (g/d)	258.44 ± 359.49	222.87 ± 78.52	230.63 ± 87.90	262.30 ± 101.59	333.68 ± 155.89	< 0.001
Vitamin A (IU/d)	1942.17 ± 4559.48	1540.54 ± 1114.21	1675.96 ± 1384.14	2169.40 ± 2441.93	3133.54 ± 3493.21	< 0.001
Beta-carotene (mg/d)	1410.24 ± 2861.50	1192.50 ± 1025.21	1316.23 ± 1285.14	1683.01 ± 1858.25	2556.12 ± 3224.31	< 0.001
Vitamin E (IU/d)	4.24 ± 5.43	3.21 ± 1.04	3.21 ± 1.03	4.13 ± 1.02	4.51 ± 2.01	< 0.001
Folate (mg/d)	314.05 ± 517.22	269.07 ± 94.51	281.54 ± 111.12	323.25 ± 137.45	413.34 ± 219.53	< 0.001
Vitamin C (mg/d)	108.34 ± 144.47	103.57 ± 41.01	112.43 ± 50.14	137.45 ± 61.06	189.42 ± 121.46	< 0.001
Vitamin B12 (mcg/d)	5.73 ± 23.28	3.72 ± 3.66	3.82 ± 4.26	5.22 ± 18.60	5.92 ± 11.95	< 0.001
Vitamin D (IU/d)	1.52 ± 2.03	1.29 ± 1.08	1.33 ± 1.11	1.52 ± 1.43	1.82 ± 1.58	< 0.001

Obtained by one way ANOVA

Table 3 presents the prevalence of hearing loss among the study participants, categorized into five quintiles based on their dietary total antioxidant capacity (dTAC) levels. The table shows the percentage of participants with hearing loss in each quintile for the whole population, as well as broken down by gender and age group. For the whole population, the prevalence of hearing loss was consistent across the quintiles, ranging from 30.8% in Q4 to 35.6% in Q3, with an overall prevalence of 33.3%. The *p*-value of 0.47 indicates that there was no statistically significant difference in the prevalence of hearing loss across the different dTAC levels for the whole population. When looking at the data by gender and age group, some variations can be observed. For men under 40 years, the prevalence of hearing loss ranged from 19.2% in Q4 to 34.4% in Q1, with a *p*-value of 0.06 suggesting a borderline significant difference. For men over 40 years, the prevalence was higher, ranging from 51.7% in Q1 to 64.2% in Q3, but the *p*-value of 0.23 indicates no significant difference. For women under 40 years, the prevalence of hearing loss ranged from 13% in Q2 to 20.9% in Q5, with a *p*-value of 0.26 indicating no significant difference. For women over 40 years, the prevalence ranged from 33.6% in Q4 to 42.2% in Q2, with a *p*-value of 0.36 also indicating no significant difference. These findings suggest that while there are some variations in the prevalence of hearing loss among different subgroups of the study participants, the differences are not statistically significant when considering the dTAC levels.

**Table 3:** Prevalence of hearing loss among study participants across quintiles of dTAC (%)

	Quintiles of dietary total antioxidant capacity (mmol/d)						*P*-value
	Q1 (*n* = 686) (≤ 8.2)	Q2 (*n* = 688) (8.2–9.15)	Q3 (*n* = 687) (9.15–10.15)	Q4 (*n* = 689) (10.15–11.7)	Q5 (*n* = 688) (≥ 11.7)	Overall	
**Whole Population**	33.31	33.43	35.62	30.82	33.52	33.35	0.47
**Men**							
< 40 years	34.41	26.89	30.31	19.23	22.45	26.74	0.06
> 40 years	51.72	54.45	64.24	54.14	57.01	56.22	0.23
**Women**							
< 40 years	17.91	13.02	15.71	15.72	20.92	16.62	0.26
> 40 years	35.44	42.23	38.62	33.63	34.95	36.93	0.36

Obtained by chi-square test

Table 4 presents the multivariable-adjusted odds ratios for hearing loss across quintiles of dietary total antioxidant capacity (dTAC), stratified by gender and age. The table provides both crude and multivariable adjusted models. The adjusted models account for factors such as smoking status, physical activity, body mass index, family history of hearing loss, hypertension, diabetes, use of ototoxic medications, and family history of ear-related trauma, accident, or infection. For the whole population, both the crude and adjusted models show no significant trend in the odds of hearing loss across the dTAC quintiles, with *p*-values of 0.68 and 0.96 respectively. When stratified by gender and age, some variations can be observed. For instance, in men under 40 years, the crude model shows a significant decreasing trend in the odds of hearing loss from Q1 to Q5 (*p*-value 0.01), which is also observed in the adjusted model (*p*-value 0.02). However, for men over 40 years, there is no significant trend observed in either the crude or adjusted models. For women under 40 years, there is no significant trend in the odds of hearing loss across the dTAC quintiles in both the crude and adjusted models. Similarly, for women over 40 years, no significant trend is observed. These findings suggest that while there are some variations in the odds of hearing loss among different subgroups of the study participants, the differences are not statistically significant when considering the dTAC levels.

**Table 4:** Multivariable-adjusted odds ratios for the hearing loss across quintiles of dietary total antioxidant capacity stratified by gender and age

Models	Q1 (*n* = 686) (≤ 8.2)	Q2 (*n* = 688) (8.2–9.15)	Q3 (*n* = 687) (9.15–10.15)	Q4 (*n* = 689) (10.15–11.7)	Q5 (*n* = 688) (≥ 11.7)	*P*-trend
	OR	OR (%95 CI)	OR (%95 CI)	OR (%95 CI)	OR (%95 CI)	
**Whole Population**						
Crude	1	1 (0.8–1.2)	1.1 (0.8–1.4)	0.9 (0.7–1.1)	1 (0.8–1.3)	0.68
Multivariable adjusted model	1	0.97 (0.8–1.2)	1.06 (0.8–1.3)	0.8 (0.6–1.05	0.9 (0.7–1.2)	0.96
**Men**						
< 40 years						
Crude	1	1.81 (1.01–3.24)	1.27 (0.68–2.36)	1.5 (0.81–2.76)	0.82 (0.43–1.56)	**0.01**
Multivariable adjusted model	1	0.67 (0.38–1.21)	0.82 (0.47–1.44)	0.48 (0.26–0.86)	0.56 (0.31–1.02)	**0.02**
> 40 years						
Crude	1	0.8 (0.51–1.27)	0.9 (0.57–1.41)	1.35 (0.85–2.16)	0.89 (0.57–1.39)	0.42
Multivariable adjusted model	1	1.2 (0.76–1.9)	1.7 (1.06–2.7)	1.1 (0.7–1.76)	1.28 (0.8–2.04)	0.43
**Women**						
*< 40 years*						
Crude	1	0.83 (0.51–1.34)	0.56 (0.34–0.95)	0.71 (0.43–1.16)	0.7 (0.42–1.18)	0.28
Multivariable adjusted model	1	0.68 (0.4–1.17)	0.86 (0.51–1.44)	0.86 (0.51–1.47)	1.24 (0.76–2.02)	0.24
*> 40 years*						
Crude	1	1.02 (0.68–1.52)	1.36 (0.92–2.02)	1.17 (0.79–1.73)	0.95 (0.63–1.4)	0.34
Multivariable adjusted model	1	1.31 (0.87–1.98)	1.14 (0.76–1.72)	0.97 (0.64–1.47)	1.02 (0.67–1.53	0.53

dTAC was adusted for energy intake by residual method

Model I: adjusted for smoking status, physical activity, body mass index, family history of hearing loss, hypertension, diabetes, use of ototoxic medications and family history of ear related trauma, accident, or infection

The *P*-value for trend across increasing quintiles of total antioxidant capacity was calculated using a multivariable logistic regression by considering the categories as ordinal variables

The strata variable was not included in the model when stratifying by itself

## Discussion

The present study, conducted on a large sample of Iranian adults, reveals a potential protective effect of a diet high in antioxidants, as measured by the dietary total antioxidant capacity (dTAC), on hearing loss in young men. The findings were established after adjusting for multiple potential risk factors. Importantly, this study marks the first investigation of the relationship between dTAC and the risk of hearing loss.

There is strong evidence that free radicals play a role in noise-induced hearing loss and the death of sensory cells [44] where previous studies support the use of antioxidants to protect the inner ear from various environmental stressors, thus preventing hearing loss. Free radicals formation can result in cell death and constricted blood flow in the cochlea which leads to a rebound increase in blood flow [45]. Animal studies have demonstrated that administering alpha tocopherol or combinations of antioxidants such as vitamins C can decrease the formation of free radicals, prevent hearing loss, and improve the health of sensory cells in the inner ear, indicating a potential preventive or therapeutic effect on hearing loss [16, 46, 47]. Moreover, cochlear redox unbalance is the main mechanism of damage involved in the pathogenesis of noise-induced hearing loss [28]. Indeed, the increased free radical production, in conjunction with a reduced efficacy of the endogenous antioxidant system, plays a key role in cochlear damage induced by noise exposure [28]. In conclusion, dietary antioxidants play a crucial role in hearing health by influencing the function of the inner ear and potentially mitigating the risk of hearing impairment.Most previous studies have investigated the association between individual or specific combination of antioxidants with hearing loss and not total dietary antioxidants. As part of the National Health and Nutrition Examination Survey (NHANES) 2001–2004 study, the cross-sectional data collected from 2592 individuals aged between 20 and 69 years, offers intriguing evidence of a synergistic impact on hearing loss from the concurrent consumption of antioxidants (beta-carotene and vitamins C) and magnesium [48]. However, in this study the authors assessed specific antioxidants and not total antioxidants in the diet which might not be totally comparable with our study. Similarly, earlier research conducted among elderly individuals in the Blue Mountains found a notable association between the intake of dietary antioxidants (vitamins A and E) and the occurrence of hearing loss. However, it was not associated with the incidence of hearing loss over a 5-year period [49] which might be due to the limited duration of the study.

On the other hand, other studies have failed to find a significant association. A study in a workplace on noise-induced hearing loss in men that looked at the amounts of vitamin A, E, B12, and folic acid in workers with hearing loss discovered no substantial discrepancy in the blood levels of vitamins A or E or folic acid between the study participants and the control group while it found a significant low levels vitamin B12 in patient group compared to the control group [50]. However, it’s worth noting that these measured biomarkers have short half-lives which may explain the insignificant findings. Similarly, a follow-up study conducted on 26,273 male individuals in the United States from 1986 to 2004 found that an increase in beta-carotene, vitamins C and E consumption did not lower the risk of developing hearing problems [25]. However, the results showed that a higher intake of total folate was linked to a decreased risk of hearing loss. In this study, hearing loss was self-reported which might have affected the results. Also, in a prospective cohort study of 65,521 women in the Nurses’ Health Study II, conducted from 1991 to 2009, it was found that a lower risk of hearing loss is associated with higher intakes of β-carotene, β-cryptoxanthin, and folate, either total or from diet, while a higher risk is associated with higher vitamin C intake [51]. However, this study has an important limitation where it used self-reported data on hearing loss.

Our study diverges from previous research by evaluating the overall dietary antioxidant capacity rather than individual antioxidants or specific combinations of them. This approach is necessary because previous studies have yielded conflicting results regarding the relationship between individual antioxidants and hearing loss, with some showing a protective effect, some showing a detrimental effect, and some showing no effect. For example, studies have found that vitamin C [51] and vitamin D [52] may increase the risk of hearing loss, while vitamin E [53] had no effect. Thus, it is crucial for future research to assess specific combinations of antioxidants, instead of individual antioxidants or the total dietary antioxidant.

It is possible that the significant association found in young men, but not in women, may be due to several factors. At a younger age, individuals are likely to have a higher intake of dietary antioxidants, which may be more effective in preventing hearing loss. On the other hand, as individuals reach the age of 40 or above, various factors such as age-related health conditions may limit the effectiveness of dietary antioxidant intake on hearing. Additionally, it’s also possible that the lack of significant association in women may be due to the tendency of females to underreport their dietary intake compared to men [54] which might have distorted the association.

The present study has several limitations that need to be acknowledged. Despite a relatively large sample size, the cross-sectional design of the study prevents us from making causal inferences between nutrients and hearing loss. Additionally, the dietary assessment based on a food frequency questionnaire (FFQ) may be subject to recall bias. We also lack information on noise exposure, which could be a crucial factor affecting the results of the study. Furthermore, the relatively small elderly population in the study could be a limitation, as hearing loss is most prevalent in this age group. It should also be noted that our study did not account for antioxidants intake from supplements, as the dietary total antioxidant capacity (dTAC) measure used in the study only considers dietary intake. Moreover, our study did not consider the potential interactions between different nutrients, which could influence their overall effect on hearing loss. The FFQ, while widely used, may not fully capture the complexity and variability of dietary patterns. Also, our study population may not be representative of the general population, limiting the generalizability of our findings. Lastly, we did not account for potential changes in dietary habits over time, which could influence the TAC and its relationship with hearing loss.

On the other hand, this study has several important strengths. To our knowledge, it is the first study to use dTAC to assess its relationship with hearing loss. The dietary assessment was conducted using a validated FFQ, hearing loss was evaluated by licensed audiologists following stringent standards, and a novel indicator of dTAC was used to assess the overall antioxidant intake from the diet. Additionally, the study accounted for several important covariates.

In summary, this study suggests that a higher intake of total dietary antioxidants may be linked to a lower risk of hearing loss in young men. However, this association was not observed in the overall population or among women. Due to the cross-sectional design of the study, it is not possible to definitively conclude that antioxidant intake causes a reduction in hearing loss risk. Nonetheless, the findings have significant public health implications, suggesting that a diet rich in antioxidants may play a role in reducing the risk of hearing loss, particularly in young men. Further research is needed to confirm these findings and explore their implications for older adults and women.

## Data Availability

Available from the corresponding author on reasonable request.

## Abbreviations

dTAC: Dietary total antioxidant capacity
FFQ: Food frequency questionnaire
FRAP: Ferric reducing antioxidant power
IPAQ: International physical activity questionnaire
METS: Metabolic equivalents
